# A Clinical Decision Support System for Assessing the Risk of Cervical Cancer: Development and Evaluation Study

**DOI:** 10.2196/34753

**Published:** 2022-06-22

**Authors:** Nasrin Chekin, Haleh Ayatollahi, Mojgan Karimi Zarchi

**Affiliations:** 1 Department of Health Information Management School of Health Management and Information Sciences Iran University of Medical Sciences Tehran Iran; 2 Health Management and Economics Research Center, Health Management Research Institute Iran University of Medical Sciences Tehran Iran; 3 Department of Obstetrics and Gynecology School of Medicine Iran University of Medical Sciences Tehran Iran; 4 Endometriosis Research Center Iran University of Medical Sciences Tehran Iran

**Keywords:** cervical cancer, clinical decision support system, risk assessment, medical informatics, cancer, oncology, decision support, risk, CDSS, cervical, prototype, evaluation, testing

## Abstract

**Background:**

Cervical cancer has been recognized as a preventable type of cancer. As the assessment of all the risk factors of a disease is challenging for physicians, information technology and risk assessment models have been used to estimate the degree of risk.

**Objective:**

The aim of this study was to develop a clinical decision support system to assess the risk of cervical cancer.

**Methods:**

This study was conducted in 2 phases in 2021. In the first phase of the study, 20 gynecologists completed a questionnaire to determine the essential parameters for assessing the risk of cervical cancer, and the data were analyzed using descriptive statistics. In the second phase of the study, the prototype of the clinical decision support system was developed and evaluated.

**Results:**

The findings revealed that the most important parameters for assessing the risk of cervical cancer consisted of general and specific parameters. In total, the 8 parameters that had the greatest impact on the risk of cervical cancer were selected. After developing the clinical decision support system, it was evaluated and the mean values of sensitivity, specificity, and accuracy were 85.81%, 93.82%, and 91.39%, respectively.

**Conclusions:**

The clinical decision support system developed in this study can facilitate the process of identifying people who are at risk of developing cervical cancer. In addition, it can help to increase the quality of health care and reduce the costs associated with the treatment of cervical cancer.

## Introduction

Cervical cancer is one of the most common and deadliest cancers after breast cancer in women [1]. Approximately 85% of cervical cancer deaths occur in transitional countries, and the rate of cervical cancer death in low- to middle-income countries is 18 times higher than that of high-income countries [2]. Among the causes of cervical cancer, human papillomavirus (HPV) types 16 and 18 are associated with more than 70% of cervical cancers. Other risk factors include early marriage, sexual intercourse before the age of 16, multiple sex partners, smoking, and some genital infections, such as HIV or chlamydia, that can be transmitted through sexual contact [3].

Cervical cancer has been recognized as a preventable type of cancer, as it has a long journey before tissue invasion, and can be prevented by proper screening plans and treating primary lesions [4]. However, risky cases are not diagnosed at an early stage in most transitional countries mainly due to the shortage of obstetricians and gynecologists or patients’ fear of and objection to invasive procedures. Therefore, most women with this disease, as compared with other diseases, die at a younger age [5]. To solve this problem, cervical cancer screening and the risk assessment of this disease are among the most common actions that should be taken with the aim of prevention, diagnosis, and treatment of lesions at the primary stage [4]. Current statistical risk assessment models estimate the likelihood of cancer development by examining the association between genetic, environmental, and behavioral risk factors [6]. These models classify women as high- and low-risk patients using clinical data. As a result, invasive procedures are not required for all patients and are only recommended for high-risk patients [7].

As mentioned before, the shortage of different physician specialties, including obstetricians and gynecologists, is among the substantial barriers to providing health care services for women in many low- and middle-income countries [8]. Therefore, a team-based care model along with using digital tools has been suggested to increase the accessibility and quality of health care services [9]. Currently, the use of information technology, and in particular, the use of clinical decision support systems (CDSSs) in the field of medicine has supported other traditional approaches to solve complex medical issues and make more appropriate decisions [10]. Simply, a CDSS is an interactive and flexible information system that is developed specifically to support solving nonstructural problems and improve the decision-making process [11]. These systems can be used by different health care professionals including general practitioners (GPs) and nurses and help them make the right decision at the point of need. The applications of CDSSs include screening different diseases, providing clinicians with reliable information for decision-making, presenting a variety of treatment strategies, and predicting drug interactions to improve patient care and reduce medical and nursing errors [12].

It is also expected that using health information technologies such as CDSS helps improve equity by providing health care services for different groups of patients in a variety of geographical locations [13]. However, there might be some shortfalls in using CDSSs. For example, human decision-makers may directly adopt computer recommendations mainly due to reasons such as increasing efficiency, the higher objectivity of computer conclusions, or having difficulty justifying any deviation from the computer recommendations. Moreover, low-quality data may cause the system to make incorrect decisions, and problems may arise when contextual factors that are relevant but not represented in the data sets are ignored in decision-making. This may also cause errors in identifying high-risk patients. Other risks of automated decisions include the shifting of responsibility, potential manipulation, and the lack of traceability by patients [13].

In the field of oncology, CDSSs can help assess the risk of cancer development by using clinical data and quantifying the impact of cancer risk factors [14]. These systems can also support early disease detection and allow GPs to provide a care plan when specialists are not available [15,16]. Although some similar systems have been previously developed for cervical cancer risk assessment, the number and types of input and output variables and the types of rules and algorithms used are different. According to the literature, machine learning algorithms to predict cervical cancer [17], artificial neural networks (ANNs) to combine the cytology and biomarker results [18], and ANNs to classify the normal and abnormal cells in the cervix region of the uterus [19] have been applied in previous studies. However, in these studies, the cytology results were the main input variables. Given the limited number of research conducted on the application of information technology to assess the risk of cervical cancer, the aim of this study was to develop and implement a CDSS to assess the risk of this disease by considering more simple variables to help patients and clinicians avoid unnecessary invasive procedures, save time, reduce costs, and increase the quality of care.

## Methods

This study was conducted in 2 phases in 2021.

### Phase 1

The first phase of the study included determining the essential parameters for assessing the risk of cervical cancer. Initially, a list of these parameters was provided based on literature reviews [4,15,20-24]. Subsequently, 20 gynecologists completed a 5-point Likert scale questionnaire (very important=5, important=4, moderately important=3, slightly important=2, and unimportant=1) to determine the most important parameters included on the list. The questionnaire consisted of 2 sections. The first section collected the participants’ personal information, such as age and work experience, and the second section consisted of 50 parameters and risk factors related to cervical cancer. The face and content validity of the questionnaire was assessed by 5 gynecologists. The reliability of the questionnaire was calculated using the test-retest method, and 15 gynecologists out of the research sample were asked to complete the questionnaire twice within 2 weeks. Afterward, the correlation coefficient was calculated for the questionnaire (*r*=.87).

To analyze the data, descriptive statistics and SPSS software (version 24; IBM Corp) were used. Initially, the mean values and SDs were calculated for each parameter. All parameters with a mean value of 4 or more were selected to focus on the main parameters and facilitate the process of writing the rules [10]. Subsequently, one of the gynecologists was consulted, and 8 important parameters were selected to be included in the system.

### Phase 2

In the second phase of the study, the system rules were written based on the findings of the first phase of the research and by using MATLAB software (version 9.5; MathWorks Inc). The graphical user interface of the system was designed, and the sensitivity, specificity, and accuracy of the system were evaluated. In this phase, the required data were collected from the outpatient medical records of patients referred to gynecology clinics (n=93). The gynecologists were requested to complete a data collection form including the 8 selected parameters for each patient and determine the patient’s risk of cervical cancer based on their own knowledge and experience. Finally, the level of the risk suggested by system was compared to the gynecologists’ opinions (gold standard) using the Cohen κ coefficient. A κ value greater than 0.75 indicates a very good agreement, a κ value less than 0.4 indicates a weak agreement, and a κ value between 0.4 and 0.75 indicates a relatively good agreement [10]. The receiver operating characteristic (ROC) curve of the system was also drawn. The greater the diagnostic power of the system, the ROC curve will be above the square diameter and closer to the ideal condition of an area under curve of 1 [10].

### Ethics Approval

Ethics approval was obtained from the National Committee of Ethics in Biomedical Research (IR.IUMS.REC.1400.940).

## Results

The findings of the first phase of the study indicated that of the 20 gynecologists, those in the age range of 41-45 years (n=8, 40%) and with work experiences of 5-10 years (n=12, 60%) were the most frequent. According to the participants’ perspectives, a number of general and specific parameters were more important than others for assessing the risk of cervical cancer (Table 1).

As previously noted, one of the gynecologists was consulted, and 8 important parameters were selected among all items with a mean value of 4 or more to be included in the system. These parameters were the history of high-risk HPV (16, 18), number of patient’s sexual partners, history of various sexually transmitted infections, smoking status, Papanicolaou (Pap smear) test results, number of husband’s legal sexual partners, age of the first sexual intercourse, and history of cervical and vaginal diseases.

**Figure 1:**
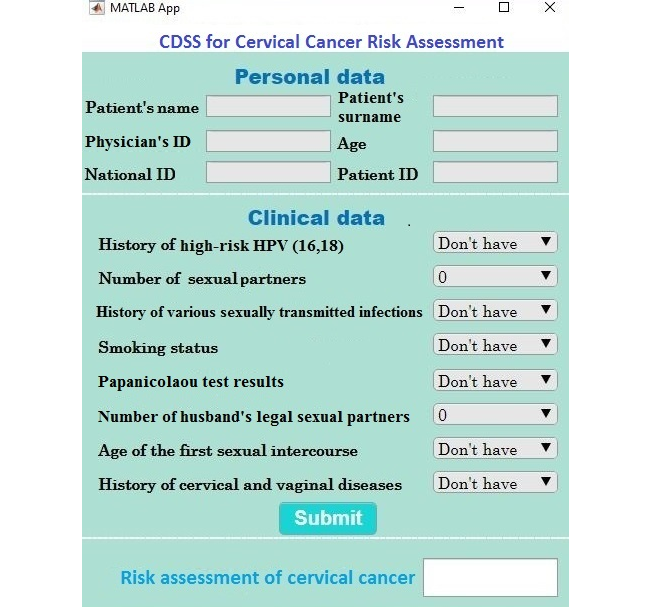
User interface of the clinical decision support system (CDSS) to assess the risk of cervical cancer. HPV: human papillomavirus.

After writing the If-Then rules, the graphical user interface of the system was designed using MATLAB software (Figure 1). The interface of the CDSS consisted of input and output variables. The input variables included the data for the 8 important parameters mentioned above, and the output variable was the risk assessment result that showed 4 different levels: safe, low risk, moderate risk, and high risk.

The system was evaluated using data collected from patients who were referred to gynecological clinics. In total, 100 patients visited the gynecological clinics in 1 month; however, 7 patients were excluded due to definite cervical cancer diagnoses, and the number of patients was reduced to 93. The patients’ data were entered into the system and the results were compared to the gynecologists’ opinions. Table 2 shows the values of sensitivity, specificity, and accuracy for the different risk groups.

**Figure 2:**
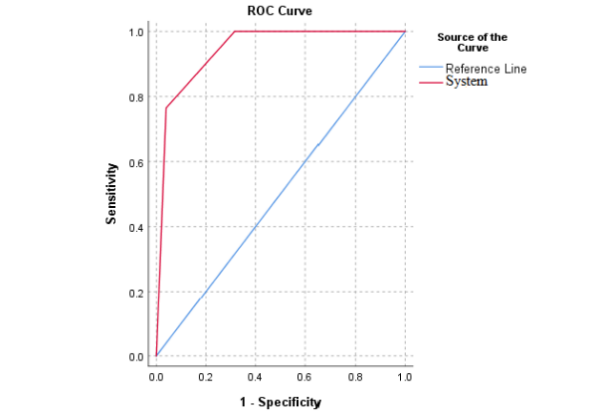
Receiver operating characteristic (ROC) curve.

The Cohen κ coefficient was also calculated to compare the risk level assessed by the CDSS and the gynecologists’ opinions. The results revealed that the κ value was 0.89 for the low-risk group, 0.73 for the moderate-risk group, 0.74 for the high-risk group, and 0.79 for the whole system. As the κ values were greater than or close to 0.75, it can be concluded that there was a good agreement between the system performance and gynecologists’ opinions. The ROC curve of the system was also drawn (Figure 2). The results showed that the ROC curve was above the square diameter and close to the ideal condition of an area under curve of 1. This indicated high diagnostic power by the system.

**Table 1:** Important general and specific parameters for assessing the risk of cervical cancer.

Parameter			Degree of importance^a^											Mean (SD)
			Very important, n (%)		Important, n (%)		Moderately important, n (%)		Slightly impor- tant, n (%)		Unimportant, n (%)			
**General parameters**														
	Patient’s age	9 (45)		8 (40)		3 (15)		0 (0)		0 (0)		4.30 (0.73)		
	Smoking status	11 (55)		5 (25)		4 (20)		0 (0)		0 (0)		4.40 (0.75)		
	History of exposure to smoke	10 (5)		5 (25)		4 (20)		1 (5)		0 (0)		4.20 (0.95)		
	Patient’s social status	8 (40)		9 (45)		3 (15)		0 (0)		0 (0)		4.25 (0.71)		
	Marital status	8 (40)		9 (45)		3 (15)		0 (0)		0 (0)		4.25 (0.71)		
	History of high-risk HPV^b^ (16, 18)	20 (100)		0 (0)		0 (0)		0 (0)		0 (0)		5 (0)		
	History of HPV vaccination	13 (65)		5 (25)		2 (10)		0 (0)		0 (0)		4.55 (0.68)		
	Family history of cervical cancer	12 (60)		4 (20)		3 (15)		0 (0)		1 (5)		4.30 (1.08)		
	Genetic factors	12 (60)		3 (15)		5 (25)		0 (0)		0 (0)		4.35 (0.87)		
	Number of sexual partners	16 (80)		3 (15)		1 (5)		0 (0)		0 (0)		4.75 (0.55)		
	Number of husband’s legal sexual partners	16 (80)		4 (20)		0 (0)		0 (0)		0 (0)		4.80 (0.41)		
	Age of marriage	7 (35)		10 (50)		3 (15)		0 (0)		0 (0)		4.20 (0.69)		
	Age of the first sexual intercourse	9 (45)		7 (35)		4 (20)		0 (0)		0 (0)		4.25 (0.78)		
	Number of sexual intercourse per month	7 (35)		6 (30)		7 (35)		0 (0)		0 (0)		4 (0.85)		
**Specific parameters**														
	Sexual health status	10 (50)		10 (50)		0 (0)		0 (0)		0 (0)		4.5 (0.51)		
	Papanicolaou test results	17 (85)		2 (10)		0 (0)		1 (5)		0 (0)		4.75 (0.71)		
	History of immune deficiency diseases	14 (70)		6 (30)		0 (0)		0 (0)		0 (0)		4.70 (0.47)		
	History of cervical and vaginal diseases	16 (80)		3 (15)		1 (5)		0 (0)		0 (0)		4.75 (0.55)		
	History of ovarian and fallopian tube diseases	7 (35)		8 (40)		4 (20)		1 (5)		0 (0)		4.05 (0.88)		
	History of hysterectomy	8 (40)		6 (30)		4 (20)		2 (10)		0 (0)		4 (1.02)		
	History of sexually transmitted infections	14 (70)		6 (30)		0 (0)		0 (0)		0 (0)		4.70 (0.47)		

^a^Very important=5, important=4, moderately important=3, slightly important=2, and unimportant=1.

^b^HPV: human papillomavirus.

**Table 2:** Sensitivity, specificity, and accuracy of the system for different risk groups.

Risk group	Evaluation criteria		
	Sensitivity, %	Specificity, %	Accuracy, %
Low risk	93.70	95.50	94.62
Moderate risk	78.26	90	87.09
High risk	76.47	96.05	92.47
Mean values for the system	82.81	93.85	91.39

## Discussion

### Principal Findings

In this study, the essential parameters for cervical cancer risk assessment were identified and divided into 2 categories of general and specific parameters. To design a CDSS, the most important risk factors were selected based on the gynecologists’ opinions and consultation with a specialist. The results of the evaluation study showed that the developed system had a high level of sensitivity, specificity, and accuracy and, in most cases, was able to identify at-risk patients similar to the specialists.

Assessing the risk of a disease is one of the greatest challenges in medical sciences. Most clinical decisions are made based on the physicians’ personal understanding and experience; however, their expertise may not be adequate for assessing the risk of all diseases or disorders. Therefore, the risk assessment of diseases has been the focus of many research studies in recent years [10]. As there are different risk factors for a disease, information technology and risk assessment models are used to quantify the risk level [21,25]. Regarding cervical cancer, it is possible to identify at-risk women by determining the risk factors and measuring the effect of these factors on the risk of cancer. In addition, prevention or intervention in the early stages of the disease can be made possible by early detection in patients and then carrying out further examinations [16,26].

### Comparison With Prior Studies

In this study, the patient’s age, smoking status, social status, and marital status were the general parameters and the history of high-risk HPV (16, 18), history of HPV vaccination, family history of cervical cancer, genetic factors, and number of patient’s sexual partners were the specific parameters that had the highest mean values of importance. Similarly, in a study conducted by Vaisy et al [27], the patient’s age, age of the first delivery, history of abortion and curettage, number of pregnancies, and economic and social status were identified as risk factors of cervical cancer. Vaisy et al also showed that marital status, the number of marriages, marriage under the age of 16 years, and taking birth control pills can increase the risk of cervical cancer.

Another study conducted by Nojomi et al [28] indicated that demographic variables such as marital status, occupation, literacy, the duration of using birth control pills, the history of abortion, the family history of cervical cancer, smoking status, age at marriage, and mother’s age at the birth of her first child are among the cervical cancer risk factors. The researchers also indicated that a positive family history of cervical cancer, low age of marriage, high number of pregnancies, low age at the birth of the first child, and long-term use of birth control pills were the most significant risk factors. Similarly, Nkfusai et al [29] examined the role of smoking status, the number of sexual partners, the family history of cervical cancer, the history of HIV infection, and having more than 5 deliveries as cervical cancer risk factors.

Therefore, the essential parameters for assessing the risk of cervical cancer found in the first phase of the study were consistent with the findings of other similar studies. It should be noted that although 2 groups of general and specific parameters were considered in this study, 8 parameters were selected based on consulting with a gynecologist to facilitate the process of rule writing, developing, and implementing the CDSS. These 8 parameters were the history of high-risk HPV (16, 18), number of sexual partners, history of various sexually transmitted infections, smoking status, Papanicolaou test results, number of husband’s legal sexual partners, age of the first sexual intercourse, and history of cervical and vaginal diseases. These parameters have also been mentioned in other similar studies [27-29]. After determining the essential parameters in assessing the risk of cervical cancer, a prototype of the CDSS was developed using MATLAB software. The rules of the system were determined after consulting a gynecologist, and the graphical user interface was developed using MATLAB software. The users could enter data into the system, and the result of the cervical cancer risk assessment would be displayed as safe, low risk, moderate risk, or high risk.

Similarly, Omololu and Adeoluo [30] extracted a number of cervical cancer risk factors from patient records. These risk factors included HPV infection, the number of sexual partners, the age of the first sexual intercourse, extramarital affairs of spouses, economic and social status, the use of oral birth control pills, and genetic history. In their study, cervical cancer diagnosis was considered as the system output and adaptive neuro-fuzzy inference was used. However, in this study, the system was able to assess the risk of cervical cancer by using If-Then rules.

After developing the system, the data collected from the outpatient medical records were used to evaluate the system performance. Among the low-risk, moderate-risk, and high-risk groups, the highest sensitivity (93.70%) and accuracy (94.62%) belonged to the low-risk group, the highest specificity (96.05%) and lowest sensitivity (76.47%) belonged to the high-risk group, and the lowest specificity (90%) and accuracy (87.09%) belonged to the moderate-risk group. In general, the sensitivity, specificity, and accuracy of the system were calculated to be 82.81%, 93.85%, and 91.39%, respectively.

Similarly, Hu et al [20] used a regression model and an ANN to assess the risk of cervical cancer. After evaluating the model, the sensitivity and specificity of the model were 95.2% and 99%, respectively. In another study, Lee et al [24] validated a risk scoring system. They used patient medical records to collect the data and the Cox risk model to determine the risk score. The results indicated that the sensitivity and specificity in the group under Papanicolaou screening with a follow-up of less than 3 years were 75% and 94.1%, respectively. The sensitivity and specificity in the similar group with a follow-up of less than 5 years were 66.7% and 93.5%, respectively, and in the screening group using cytological tests, the sensitivity and specificity were 88.2% and 87.7%, respectively. Bountaries et al [31] used the retrospective data of patients who underwent colposcopy. Their CDSS classified cancer lesions using a hybrid genetic algorithm and Bayesian classification. To evaluate the system, they compared the sensitivity and specificity of their CDSS in diagnosing cancerous lesions with the Papanicolaou test and HPV detection results. The sensitivity and specificity of the developed system were 83.4% and 88.1%, respectively.

It is notable that the sensitivities, specificities, and accuracies cannot be compared between the different systems mainly due to the differences in the input and output variables and algorithms used. Although neural networks and other algorithms that might have higher precision in detecting at-risk patients were not used in this study, the results of this study showed that the developed system had a high level of sensitivity, specificity, and accuracy similar to other systems and could be used to screen and identify women at risk of developing cervical cancer. The designed system can be used by different health care providers including nurses, GPs, and gynecologists, as it had been developed based on basic clinical data. It can help regular screenings and prevent invasive tests for all patients. Moreover, identifying at-risk women at the early stages of the disease can help treat primary lesions and reduces malignancy and death [16]. In addition, a better allocation of heath care resources and improving the quality of care are expected by classifying patients into different risk groups.

### Research Limitations

There are various parameters to assess the risk of cervical cancer; however, it is difficult to gather and consider all of these parameters in a single CDSS. Therefore, in this study, the essential parameters were selected and considered for developing the system based on the gynecologists’ opinions. Including other parameters in future systems and using more sophisticated methods for system design may help assess the risk of cervical cancer more precisely. Future researchers can use parameters that were not included in the current system, or they can use new parameters that might be introduced by other researchers.

### Conclusion

The aim of this study was to develop a CDSS to assess the risk of cervical cancer. In this study, 8 essential parameters were selected and considered as input variables. The output of the system showed the risk of cervical cancer in 4 levels: safe, low risk, moderate risk, and high risk. The findings of this study revealed that the system performance was very similar to the gynecologists’ opinions. Such a system could be used for cervical cancer screening or in regions where access to gynecologists is limited. The use of this system can help improve the quality of care and manage patients more effectively. Moreover, the reduction of the mortality rate of cervical cancer through continuous and timely patient screening would be another benefit of using this system.

## Abbreviations

ANN: artificial neural network
CDSS: clinical decision support system
GP: general practitioner
HPV: human papillomavirus
ROC: receiver operating characteristic
